# Enhanced Modbus/TCP Security Protocol: Authentication and Authorization Functions Supported

**DOI:** 10.3390/s22208024

**Published:** 2022-10-20

**Authors:** Tiago Martins, Sergio Vidal Garcia Oliveira

**Affiliations:** 1 Departamento de Engenharia Elétrica, Universidade do Estado de Santa Catarina, Joinville 89219-710, Brazil; 2 Departamento de Engenharia de Telecomunicações, Elétrica e Mecânica, Universidade Regional de Blumenau, Blumenau 89030-000, Brazil

**Keywords:** cybersecurity, Modbus, Zero Trust, IIoT, industry 4.0, automation, access control, RBAC, IEC 62443

## Abstract

The Zero Trust concept is being adopted in information technology (IT) deployments, while human users remain to be the main risk for operational technology (OT) deployments. This article proposes to enhance the new Modbus/TCP Security protocol with authentication and authorization functions that guarantee security against intentional unauthorized access. It aims to comply with the principle of never trusting the person who is accessing the network before carrying out a security check. Two functions are tested and used in order to build an access control method that is based on a username and a password for human users with knowledge of industrial automation control systems (IACS), using simple means, low motivation, and few resources. A man-in-the-middle (MITM) component was added in order to intermediate the client and the server communication and to validate these functions. The proposed scenario was implemented using the Node-RED programming platform. The tests implementing the functions and the access control method through the Node-RED software have proven their potential and their applicability.

## 1. Introduction

The Industrial Internet of Things (IIoT) enables the development of factories, electrical grids, and other intelligent systems, which creates market opportunities for equipment manufacturers, internet providers, and software developers. In the coming years, sensors, machines, objects, and IIoT devices will all be connected, generating 45% of all internet traffic. Of these, 37% will be generated by things in the manufacturing area and 7% will be generated by electricity-related things [1,2].

An IIoT system connects the industrial control systems with analytical, enterprise, and autonomous systems. By optimizing the operation and enabling the control, the collaboration, and the decision making, autonomous equipment and business processes can evolve manufacturing into a new industrial era, which is known as the Industry 4.0 [3].

With Industry 4.0, industrial devices are increasingly connected to the internet and corporate intranet in order to provide real-time information for their applications and users, and, therefore, are consequently more exposed to cyber threats [4].

Operational technology (OT) systems differ from traditional information technology (IT) systems because they use sensors and actuators in industrial environments. These interact with the real world, in which uncontrolled changes can generate dangerous situations in the field. This potential risk elevates the importance of these systems’ security, reliability, privacy, and resiliency above the levels that are expected in many traditional IT environments [3].

Being one of the pillars of Industry 4.0 [5], cybersecurity is embryonic for IACS (industrial automation and control systems). With the digital transformation, the industry started a race to offer digital services to its customers, in which the equipment supporting legacy communication protocols, such as Modbus, which until then were isolated, began to be exposed in order to offer services such as real-time monitoring, remote’s startup, and assistance [6]. This ability to remotely control the two-way flow of information has many advantages but makes systems vulnerable to cyber-attacks. 

In the OT domain, concepts such as availability, integrity, and confidentiality (AIC) define cybersecurity and differ from IT, as the availability is more critical than the data confidentiality [3]. 

State-of-the-art research presents individuals as the main risk of compromising cybersecurity in OT [7,8] because the vast majority of industrial communication protocols were not designed to ensure the cybersecurity of the communication between devices, making the access control mechanisms in industrial devices limited and unable to employ role-based access control (RBAC) concepts and protocols with authentication and authorization capabilities. 

Modbus is a client-server application protocol that allows communication between millions of automation devices. Nevertheless, unfortunately, it lacks basic security mechanisms, which leads to several vulnerabilities. When it is correctly used, because it does not allow for the construction of robust access control, it allows the alteration of registers, either mistakenly or maliciously, by intruders or simple system operators. Furthermore, it is a protocol that lacks encryption, authentication, and authorization functions. It allows its exploitation by malicious software or unauthorized users (hackers), as it can easily be intercepted and altered in the middle of communication (man-in-the-middle attack), sending malicious (false command injection), malformed (false access injection), or delayed (replay attacks) data packets that lead to a denial of service (DoS) of the system [9].

The Zero Trust concept [10] is disseminated in IT. At the same time, it is not uncommon in the OT domain for people to have more access privileges than are necessary for their work, due to the low quality of access segmentation that is available by the current mechanisms that are applied to industrial devices [7]. In the Zero Trust Model, security professionals must eliminate the idea of untrusted networks and trusted networks (internal networks), as it considers all network traffic to be untrusted and upholds the following three fundamental principles: all entities are untrusted by default, the least privilege access is enforced, and comprehensive security monitoring is implemented [10,11].

Therefore, this work aims to improve the industrial Modbus protocol in order to enable the creation of access control mechanisms in industrial devices that allow individuals to remain to be the main security risk for the IACS. In order to achieve this, the validation of the feasibility of performing effective access control for human users is needed. It is based on a username and a password, against access attempts and casual unauthorized use, using simple means, a low level of resources, and low motivation, for IACS that provide human user input interfaces, such as HMI or SCADA systems, that are connected through industrial Ethernet networks through the protocol.

The influential authors in the automation area argue that, instead of raising the cyber-security maturity of the Modbus protocol, organizations should invest in new technologies that provide security by design, such as OPC-UA, for the protocol replacement [12]. However, because it is widespread in the industry, the option to provide security to the protocol is a solution that many organizations are likely to adopt [13].

Stuxnet [14], which was the first malware to successfully attack an industrial control system (ICS) in 2010, infected Windows-based computers on any control or SCADA system; it was used to disrupt the uranium enrichment process at the Iranian nuclear facility in Natanz. Since Stuxnet, many works have been published in order to raise the Modbus protocol’s cybersecurity maturity. A solution using integrity mechanisms through the SHA2 algorithm, authentication through RSA, and non-repudiation and anti-replay through the timestamp is presented in [15]. Another proposal that is based on SCTP (stream control transmission protocol) and HMAC (hash-based message authentication code), which is called ModbusSec, is presented in [16]. However, both of these works do not consider the confidentiality requirement for Modbus messages, as they are usually implemented using cryptography, which is expensive and presents a considerable overhead that can affect the performance in real-time communication [15,16].

Assertions are no longer true because of the advancement of technology and the possibility of using electronic components that provide encryption based on hardware such as TPM (trusted platform module) [17]. The cost is no longer a problem, and its use became viable for communication, as presented in [18], in which the author applies the TLS (transport layer security) protocol as a solution to Modbus security problems and presents experimental results that reveal the negligible impact for security applications in power grids. Additionally, in [19] the Modbus-S protocol is proposed, together with the addition of a security gateway to IACS, which was designed based on the original Modbus TCP protocol, which uses a symmetric key algorithm in order to guarantee data confidentiality and also ensures data uniqueness through a hash algorithm-based synchronization mechanism, integrity through a digital signature algorithm, and function code abuse through a “White List” filtering mechanism that is used to manage the function codes. These have been confirmed by the Modbus organization itself, which in the same year adopted the TLS protocol in place of the TCP (transmission control protocol) protocol in order to support this new reality in the industry through the publication of the new protocol specification.

Named Modbus/TCP Security, this new variant guarantees the integrity and the confidentiality of the session that is established between the client device and the server through PKI (public key infrastructure) and also proposes the transmission of RBAC (role-based access control) information using X.509v3 certificate extension [20]. The resources and new functionality are the objects of study by [13], which seek to contextualize the construction of the RBAC model through applying such specifications.

However, our understanding is that such an approach has limitations regarding the identification and the authentication of the user who performs the procedures through the interfaces that allow the iteration of human users, as they do not guarantee non-repudiation since it is not possible to validate who owns the certificate in cases where the secure session is established between IACS system components.

It is crucial to validate whether it is possible to enhance the Modbus/TCP Security protocol with authentication and authorization functions in order to build access control and enforce the least privilege for human users. In particular, once a secure and encrypted session is established between the client and the server device, as specified by the protocol, we need to identify and authenticate all of the users that make requests to the server device, whether they are humans, processes, or systems. Our approach was to build an authentication function, through user-defined functions that are provided by the Modbus protocol, in order to identify, authenticate, and return an authenticator for the given system user. A second user-defined function is built in order to encapsulate the original request and to carry out the authorization control. The authentication enables the identification of the user and the segmentation of accesses by the device. This is presented in the Enhanced Modbus/TCP Security Protocol chapter, together with a brief explanation of the Modbus protocol. This was followed by our proposal to implement the new functions in order to build an access control method for human users in compliance with the first security level of the IEC 62443-4-2 standard [21]. In order to evaluate our implementation proposal, we will deploy it on the Node-RED programming platform [22] to finally provide the tests, the discussion of the results, the conclusions, and the future research lines.

## 2. Other Related Works

Studies on the smart grid concept cover encryption, intrusion detection, prevention, privacy, and trust. In [23], a literature review that is related to trust in smart grid is presented. The definitions of trust guided this categorization according to the literature and NIST’s priority areas and conceptual domains. A new trust model for substations that detects attacks inside of the substation is also presented and tested. Using the model, the authors test the work on two publicly available datasets using three types of tests. External testing is where purely rogue devices (non-compromised substation devices) are not considered to be part of the substation network. The second is the internal test, where all devices are considered to be part of the substation network. The final test is the internal test with the IP–MAC block that takes the second test position but blacklists any device that sends a malicious message.

The intrusion detection system (IDS) for smart grid is presented in [24,25]. In the first study, an anomaly-based system called ARIES (smart grid intrusion detection system) can efficiently protect smart grid communications by combining three detection layers that are dedicated to recognizing potential cyber-attacks and anomalies against network flows, Modbus/TCP packets, and operational data. The second study proposes an IDS called MENSA (anomaly detection and classification) that adopts a new autoencoder-generative adversarial network (GAN) architecture to detect operational anomalies and classify Modbus/TCP and Distributed Network Protocol 3 (DNP3) cyber-attacks. The implemented anomaly detection and classification model detected thirteen cyber-attacks on Modbus/TCP, five cyber-attacks on DNP3, and possible anomalies that were related to operational data (i.e., time series electricity measurements). Moreover, its efficiency was validated and evaluated in a smart grid laboratory, a substation, a hydroelectric plant, and a power plant.

Industrial communication is characterized by high regularity, while cyclicality creates another dimension in order to study deviations. These resources are used by [26] in order to present a method for detecting anomalies in cyclic communication using the Modbus protocol.

Production systems are not ideal or not available for such an assessment due to the possible impacts and disruptions. The authors of [27] propose a structure called the virtual operational technology network (VOTNet), which consists of a power system, a process network, a communication network, an automation network, and a business network for cybersecurity assessment in the automation of security systems energy.

In [28], the authors developed and validated a test bench for conducting cybersecurity assessments in nuclear power plants. The approach allows the simulation of several cyber-attack scenarios against a simulated nuclear power plant (NPP) communicating with its supervisory system (SCADA/HMI) through the Modbus/TCP protocol. It also demonstrated how to use the environment to generate the datasets that are needed for intrusion detection studies.

Finally, in [9], a framework is developed to prevent false command injection, false access injection, and replay attacks in the Modbus protocol in SCADA systems. The framework was developed with a frame filtering module in order to protect the PLC against unauthorized access attacks and a second module was developed to detect replay attacks in the Modbus protocol.

## 3. Enhanced Modbus/TCP Security Protocol

The most used and disseminated information security standard in the world is ISO/IEC 27001:2013 [29], which is considered to be an international reference for information security management, just as ISO 9001 is the international reference for information security management quality. However, when it comes to cybersecurity for industrial automation and control system (IACS) devices, the IEC 62443-4-2 standard must be considered.

The standard consists of the following seven fundamental requirements: (a) The identification and authentication control specifies that all users (humans, software processes, and devices) must be identified and authenticated before accessing IACS. (b) The use control specifies that once the user is identified and authenticated, the device must be enabled to restrict the allowed actions to its authorized use, to protect itself from unauthorized actions, and to verify that the necessary privileges have been granted before allowing a user to perform the actions. (c) The system integrity seeks to prevent unauthorized manipulation or alteration of the device before and during its operation. (d) The data confidentiality guarantees the confidentiality of data that are transmitted through the communication channels and stored in the device’s memory, preventing the dissemination of unauthorized information. (e) The restricted data flow seeks to segment IACS through zones and conduits in order to limit unnecessary data flows. (f) The timely response to events specifies requirements in order to ensure the response to security breaches, enabling the notification of competent authorities and evidence of the breach so that corrective and timely actions can be taken when incidents occur. (g) The resource availability specifies requirements in order to ensure the device availability and, consequently, IACS against degradation or denial of essential services. Each fundamental requirement has several conditions that must be met depending on which of the four specified security levels is desired for the given IACS device [21].

The access control must be built on a solid foundation in order to ensure device resource availability to all of its trusted users. It ensures integrity and confidentiality through the device’s root of trust (RoT) with the secure storage of identity, cryptographic keys, and firmware validation during its boot process and with the cryptography of the data that are transmitted or stored by it. This control should be based on the concept of authentication, authorization, and accounting (AAA) [30]. Once the user is both identified and authenticated, an authorizer mechanism must control the access rights of each user to the component, enabling the asset owners and the system integrators to define the privileges of each role that is to be assigned to each user (human, software process, or device), which is authorized in the drive.

**Figure 1:**
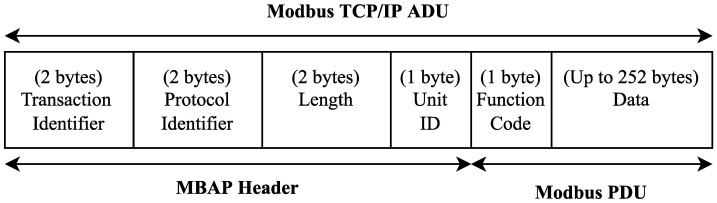
Modbus application data unit structure.

The Modbus/TCP protocol can be found in [31,32]. A TCP Modbus frame (Figure 1) consists of an MBAP (MODBUS application protocol) and a PDU (protocol data unit) and is known as an ADU (application data unit). The MBAP is a header with the same structure with seven bytes for all of the frames, while the PDU is the function requests and responses. It is limited to 253 bytes, where the first byte is designed to function code (FC), and the others are the data. There are two kinds of function codes, which are as follows: public function codes, which are specified by the protocol, and user-defined function codes, which are specific functions that are developed by users and manufacturers to support product features.

This work proposed to develop and adopt two new user-defined functions (authentication and authorization) in order to provide both capabilities and to deploy access controls in order to enhance the Modbus/TCP Security protocol.

### 3.1. Authentication User-Defined Function

**Figure 2:**
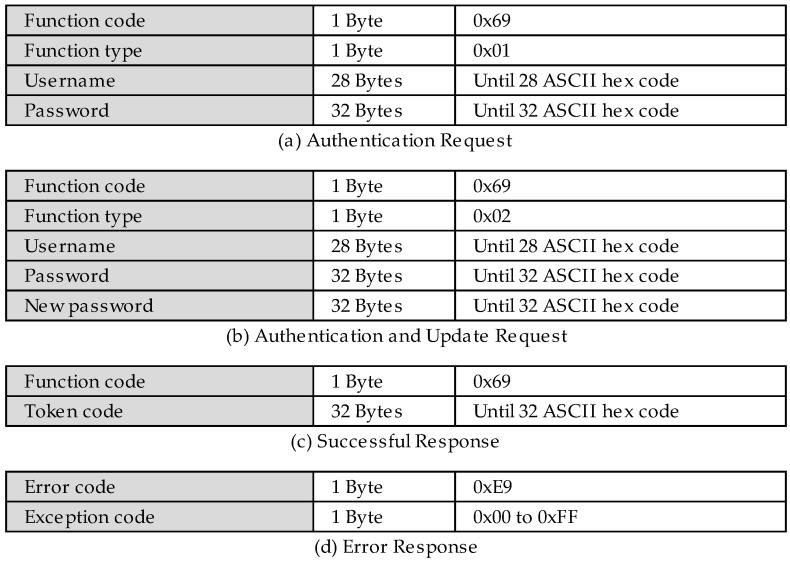
PDUs of the new authentication user-defined function.

The PDU structure of the new authentication function is presented in Figure 2. It receives the function code 0x69 and has a subsequent byte that is used to determine the type of the function, which can be any of the following functions. 

#### 3.1.1. Authentication

The byte 0x01 determines that the function will be used by a common authentication request (Figure 2a). It is followed by 28 bytes of the username and 32 bytes of the password, in which each character (ASCII table code) occupies one byte of the function data.

#### 3.1.2. Update

The byte 0x02 determines that the function will be of the update and the authentication type (Figure 2b). In addition to the username and the password, the request will inform a new password of 32 characters in order to update the current password before authenticating the user.

Figure 2c presents the PDU struct of successful responses for both types of requests. It is the function code byte as standard by Modbus, followed by a token code of 32 bytes, which is used in order to identify the authenticated user. This token code must be unique in order to guarantee the integrity of the username, for example, a SHA256 of a robust random number generation [33,34].

In case of failure during the authentication process with the new function, the server will respond to the request that was made by the client with an error message (Figure 2d). The user-defined exception code, for example, an invalid password exception, will receive the code 0x28 to inform the client that the hash code is not authenticated.

### 3.2. Authorization User-Defined Function

The newly developed function encapsulates the PDU of the original Modbus request that was made by the client in its header. 

The token is received by the authentication process, enabling the authorizer to identify which username made the respective request.

**Figure 3:**
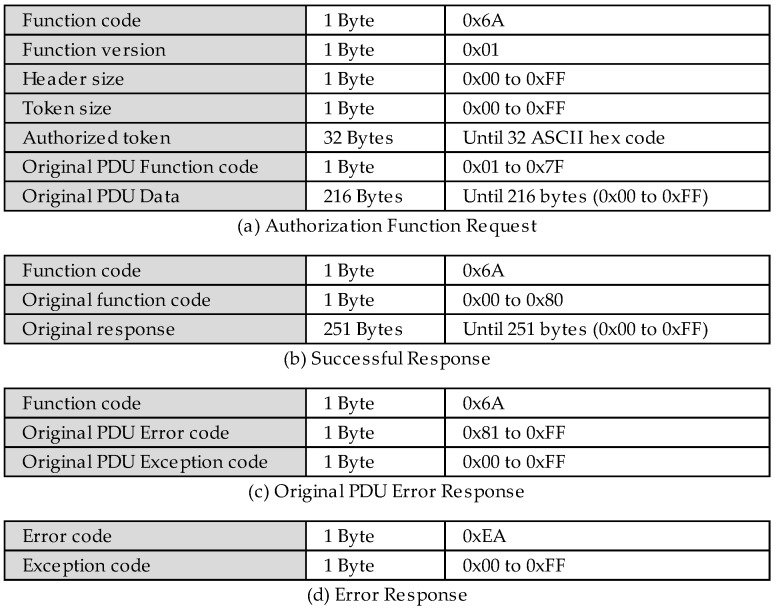
PDUs of the new authorization user-defined function.

Its PDU structure is present in Figure 3, which is known as the authorization function. For the availability, it receives the function code 0x6A. In addition to the function code, it has a byte to inform its version for future updates, followed by the header size, which helps to identify the original function’s beginning, the token’s size to facilitate the process of extracting the token, the user’s own token of 32 bytes, and finally ending with the PDU of the original request, which is limited to 216 bytes.

The struct of a successful response is present in Figure 3b. The first byte is the owner function code, followed by the original function code and the response. As the authorization function encapsulates the original PDU, if an exception occurs for the original function, or if the user does not have access permission for the given register, the function will return this exception encapsulated in a successful response frame that is performed by the new authorization function (Figure 3c). In case of failure during the authorization process with the new function (Figure 3d), the server will respond to the request with an exception message, for example, no access exception, code 0x29, in order to inform the client that token code is not authenticated.

## 4. Implementation

**Figure 4:**
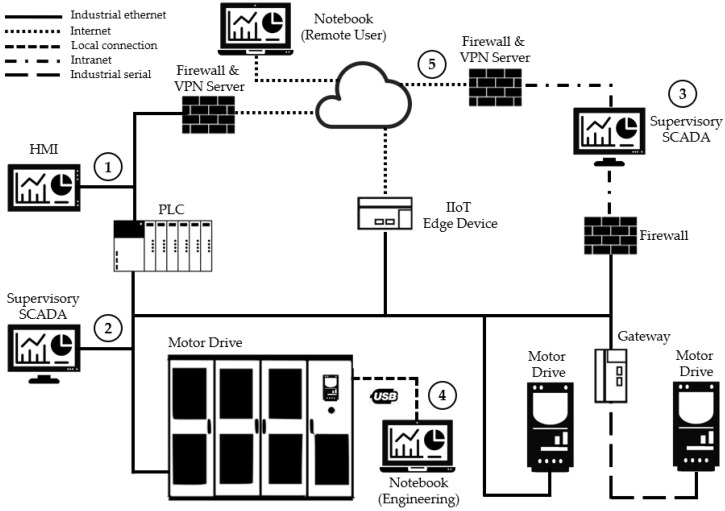
Example of an industrial automation and control system.

An IACS is represented in Figure 4, and the solid green line represents an industrial ethernet network (OT domain). As can be seen, it has a connection to the intranet in the IT domain (the solid red line) and the internet (the dashed blue line) through a firewall and IIoT edge devices. The components that allow human users to interact with the system are also represented.

Item one represents a human–machine interface, which is a graphical user interface that allows interaction between a human operator and components of IACS. It generally displays specific information about the system to the operator and can be used to change the operational or the engineering setpoints and the parameters in the system. Items two and three represent the supervisory software that are installed on computers that allow high-level visibility and interaction of the human users with the system. The first is directly connected to the industrial network, while the second is connected to the corporate network, which is usually located in the control and command centers of the production process. Item four represents the direct connection of a notebook to an IACS device, which usually happens when it is necessary to use specific software for the parameterization of the device. Finally, item five represents the remote connection to the supervisors and the devices—a common scenario when specialists need punctual intervention on IACS by specialists for corrective maintenance, with the objective of explaining and validating the applicability of the functions that are developed for the construction of systems that control the access of human users to the reading and writing of Modbus registers in IACS components. 

**Figure 5:**
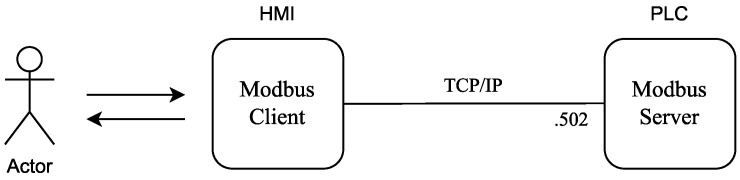
Standard Modbus client-server scenario.

Firstly, it is considered to be a minimalist system that contains only one Modbus server (PLC), receiving requests for writing and reading registers from a Modbus client (HMI). The communication between the two devices is carried out through the TCP protocol on port 502 (Modbus/TCP Standard). In this first scenario (Figure 5), there is no control over the client’s requests, therefore, the Modbus server will successfully respond to all of the public requests that are made by the Modbus client for valid registers on the server.

**Figure 6:**
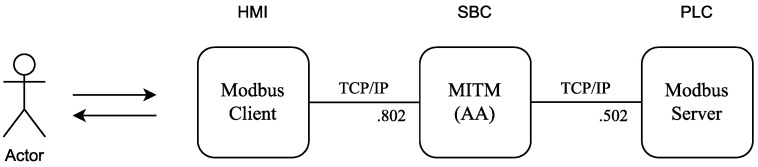
Laboratory’s scenario, MITM agent.

The proposed scenario to validate these functions considers the addition of a new component to the system (Figure 6). The man-in-the-middle (MITM) component will be responsible for intermediating the communication between the client and the server and intercepting requests to carry out the authentication process and to access control to the Modbus server. In this new scenario, instead of connecting directly to the Modbus server, the Modbus client is connected to MITM on TCP port 802. This is possible with the use of a third component with computational resources, such as single-board computers (SBC), and it is valid to improve brownfield devices with serial communication or earlier versions of the Modbus protocol. However, the idea is to embed the authentication and authorization capabilities directly into the PLC in order to be compliant with IEC 62443-4.2 and to prevent these controls from being bypassed.

Although we are exemplifying both of the scenarios with real devices, the proposed scenario was simulated using the Node-RED programming platform in this work. It is a flow-based programming tool that integrates the OpenJS Foundation. Initially developed by IBM’s emerging technology services team, it is a low-code, visual representation programming model that describes the behavior of an application as a network of nodes, where each node has a very well-defined purpose. It receives data, processes it, and then returns it to the network. It is responsible for the data flow between the nodes [22].

**Figure 7:**
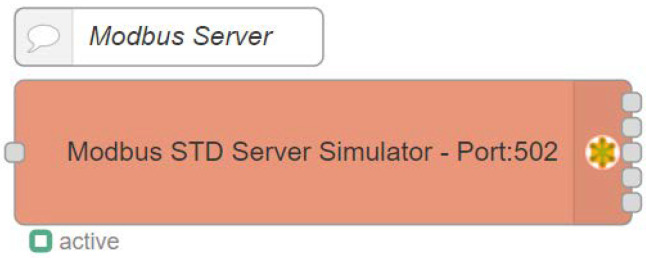
Node-RED Modbus server flow simulates the PLC.

Figure 7 shows the Modbus server flow. With a single node, the first flow that was deployed for this experimentation turned a Modbus STD server simulator available. It is a node that simulates a Modbus server. In order to configure the server, it is necessary to determine how many coils, holdings, and discrete inputs will be available to the system and to set an address and a port for the network communication. For the access control implementation maintaining the Modbus/TCP standard specification, the IPv4 localhost and the TCP port 502 were chosen.

**Figure 8:**
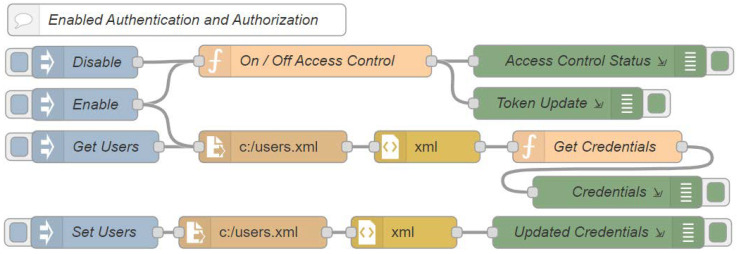
Node-RED auxiliary flow.

Figure 8 shows the flow with auxiliary functions that enable and disable the access control and configure and obtain the authorized users’ credentials in order to make requests in the system. The disable and enable nodes are of the inject type and make it possible to initialize the payload value in the flow. Both connect to the on/off access control node of the function type, which allows the execution of the previously programmed functions through the JavaScript language. This node enables or disables the system’s access control, and its output is connected to the access control status and the token update nodes, which are both of the debug types that are responsible for displaying the properties of the selected message in the debug window. In addition, the enable node is connected to the C:/users.xml node, which is a read file type that is responsible for reading the files from the running system. This node will search for the file in which the user’s credentials are stored, passing through the XML node, which is responsible for converting the file in XML format into a JavaScript object. This object is passed through the get credentials node as a message payload in order to be transmitted by the network. Then, the credentials that are stored in this JavaScript object are defined as global variables that are accessible by any nodes or flows in the system. A similar process is performed by the set users node, which allows new users and credentials to be updated and stored for the system.

**Figure 9:**
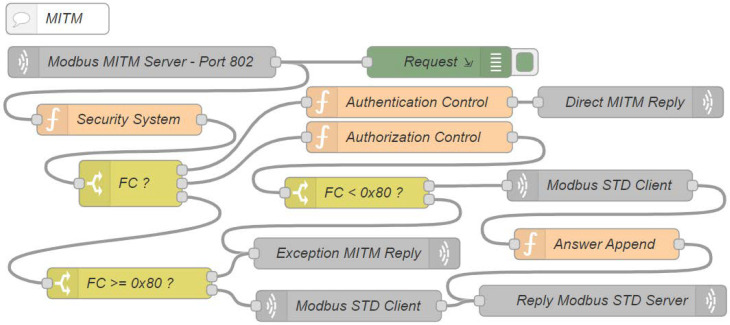
Node-RED man-in-the-middle flow simulates the SBC.

The following flow, called MITM (Figure 9), is responsible for performing the authentication and the user access control to the Modbus server through the newly developed user-defined functions.

The node Modbus MITM server port 802 is a TCP in node type and it receives all of the connection requests on the 802 TCP port. It is directly connected to the following two other nodes: the request node, which is responsible for registering all of the requests in the flow; and the security system node, which evaluates whether the access control has been activated through the auxiliary flow.

If it is disabled, all of the requests are forwarded to the third output of the FC? node, which is directly connected to it. This node is of the switch type and it switches messages according to some of its properties.

For the system under analysis, the function code of the request is evaluated when the access control is disabled, and a non-public request arrives, so the function code is replaced by its respective exception code. In Modbus it is equivalent to the function code added to the value 0x80 and forwarded to the next node, FC >= 0x80?, which parses whether the message is a request or an exception. For cases where the received message is an exception, it will forward to the exception MITM reply node, which is a TCP out reply node type that is responsible for responding to requests for its respective sources. Although, if it continues to be a request message, it is forwarded to the Modbus STD client node, which is a TCP request node type that is responsible for making requests to the Modbus server, waiting for the responses to finally be forwarded by them to the reply Modbus STD server node, and to return to their respective source, regardless of whether it is a successful or exceptional response.

However, once the access control feature has been enabled, the FC? node will have three possible outputs, as follows: the first is used for the new user-defined function of the authentication type, function code 0x69, the second is for the new authorization type user-defined function, function code 0x6A, and the third is for exceptions that are generated by the security system node, because once the access control is enabled, all of the public functions that are requested directly to the MITM will be blocked by it and their respective exception messages generated by it.

The authentication requests are sent to the authentication control node. This node is responsible for verifying the user and the password, returning the token in the case of success (Figure 2c), or exception in the case of authentication failure (Figure 2d) through the direct MITM reply node. The token is the SHA256 result of the concatenation between the timestamp and the username.

The authorization requests are forwarded to the authorization control node. This node first analyzes whether the token that is informed by the request (Figure 3a) has expired or if it is not authenticated in the system. The exception code corresponding to the error that is found is generated for these cases. However, if no problem is found, then it is evaluated whether the given user is authorized to perform the given request. In negative cases, an exception is raised, but for positive cases, the original function is decapsulated to proceed with the request. As can be seen, this node is connected exclusively with node FC < 0x80?, which evaluates whether the response from the authorization control node is an exception. If so, it forwards the message (Figure 3d) to the exception MITM reply node, which returns the exception to its respective source, but if it is not an exception, it will forward the request to the Modbus STD client node, which is responsible for performing the original requests to the Modbus server on the TCP port 502, but now, different from non-encapsulated public requests, the return that is received from the Modbus server, regardless of being a success (Figure 3b) or an exception (Figure 3c), must be re- encapsulated before returning to its original source.

**Figure 10:**
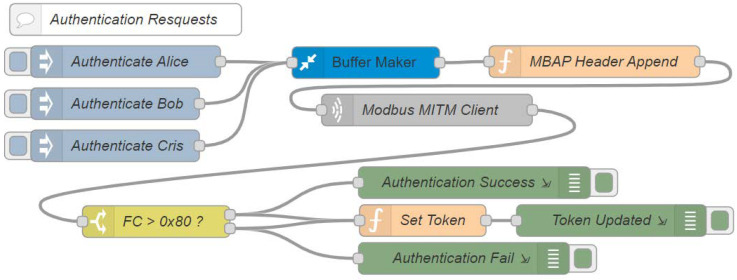
Node-RED authentication request flow simulates the first part of the HMI.

The authentication request flow is responsible for performing the user authentication requests. As can be seen in Figure 10, there are three inject nodes, one for each user in the system. Table 1 presents the permissions of each user.

When activating one of these nodes, as they are all connected to the buffer maker node, the first process to be performed is to transform the message payload into a buffer, then the MBAP header append node adds the data from the MBAP header of the previous Modbus protocol in order to send the request to the MITM flow through the Modbus MITM client node. It is the return of a given request that is sent to the FC > 0x80? Node that will validate whether the response is an exception or not. In case of an exception, the message is sent to the authentication fail node and, in parallel, this forces the global variable token to be null through the set token node. Nevertheless, if the response is successful, the authentication success node is logged and the token variable with the value is received in the response.

**Figure 11:**
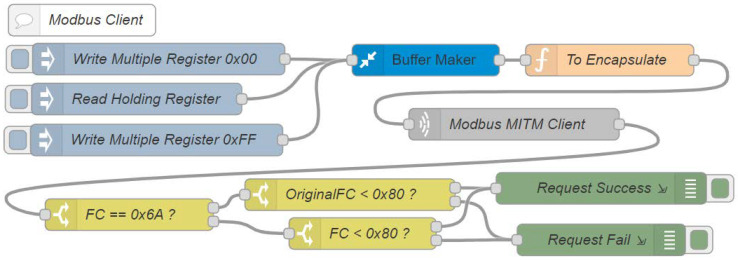
Node-RED Modbus client flow simulates second part of the HMI.

In the last system flow, Modbus client (Figure 11) is responsible for reading and writing requests to the register 0x64 from the Modbus server. The node read holding register performs a reading request in order to register 0x64 through the Modbus read holding register (0x03) function. The node write multiple register 0x00 writes the value 0x00, and the write multiple register 0xFF performs the writing request of the value 0xFF to the same register through the write multiple registers (0x10) function of the Modbus protocol.

All of the inject nodes are directly connected to the buffer maker node so that the payload is transformed into a buffer. The to encapsulate node performs the encapsulation of the original function in the new authorization function, according to Figure 3, when the access control is enabled. If it does not, it sends the original function to the Modbus MITM client node, which is responsible for sending the requests to the MITM flow. The response to these requests is sent to the FC == 0x6A? node, which responsible for directing the type of request, the original or the authorization, to its given subsequent nodes. The OriginalFC < 0x80? node evaluates whether there was an exception in the response of the original request that was made through the new authorization function (Figure 3c). The FC < 0x80? node already has the same function; however, there are exceptions from both of the original functions and the authorization function, in case the given user does not have writing access, for example (Figure 3d).

## 5. Tests

The tests that are presented in this section aim to facilitate the understanding and to validate the applicability of the two new user-defined functions for developing a method that provides authentication and authorization resources to the Modbus/TCP Security protocol. All of the values that are present on these tests are in hexadecimal [18].

Bear in mind that this work proposes improvements in the protocol, and the encryption is essential for the operation of the proposed mechanisms, because it guarantees the confidentiality of the transmitted credentials and tokens. This was chosen not to be applied in tests to encrypt the transmitted data once it was understood that its inapplicability does not affect the final results of the proposed tests, as it aims to validate the use of the two new authentication functions and to authorize users to make requests of writing and reading in the Modbus server registers.

**Figure 12:**
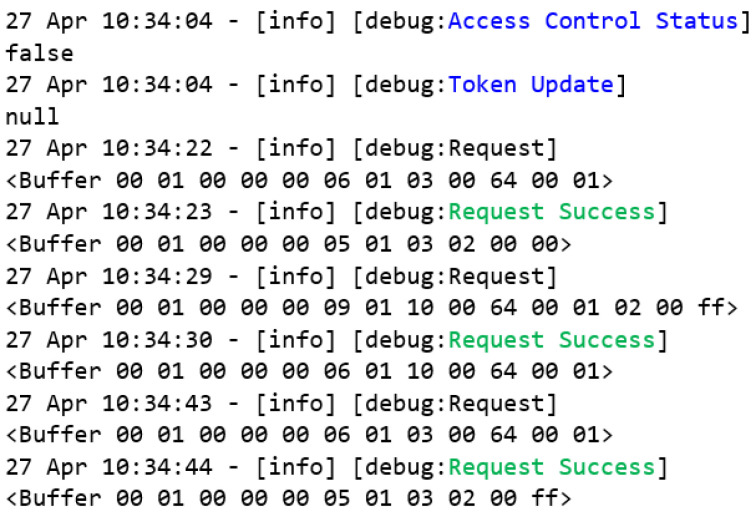
Event log of access control disabled.

As can be seen in Figure 12, the tests started with the access control deactivated. All of the requests that reach the MITM flow will be forwarded to the Modbus server.

**Figure 13:**
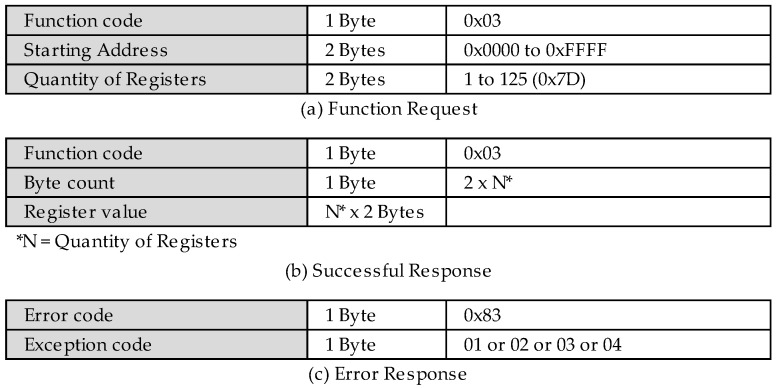
Read holding register PDUs [32].

As the access control is disabled, the token is forced to a null value. Furthermore, the read and write requests to the register 0x64 can be successfully performed through the Modbus client flow. Remember that a Modbus/TCP frame is named ADU (Figure 1). Therefore, the first request is composed of the MBAP header and the read holding register PDU (Figure 13a), and the subsequent frame is the successful response (Figure 13b), where the register value 0x00 was successfully received.

**Figure 14:**
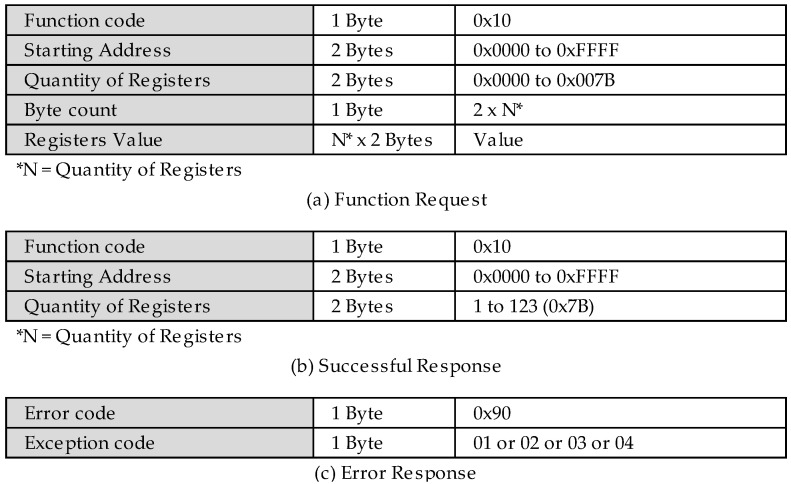
Write multiple register PDUs [32].

Then, a writing request of the value 0xFF is carried out through the function write multiple register (Figure 14), ending with a new reading that proves that the register’s value changed from 0x00 to 0xFF.

**Figure 15:**
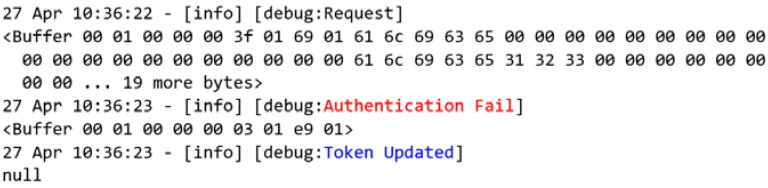
Event log of new function with access control disabled.

Continuing with the access control deactivation, a request is made using the new user-defined function for user authentication. The results are shown in Figure 15. Moreover, the MITM flow forwards this request to the Modbus server that does not understand the requested function, returning an exception, according to Figure 2d.

**Figure 16:**
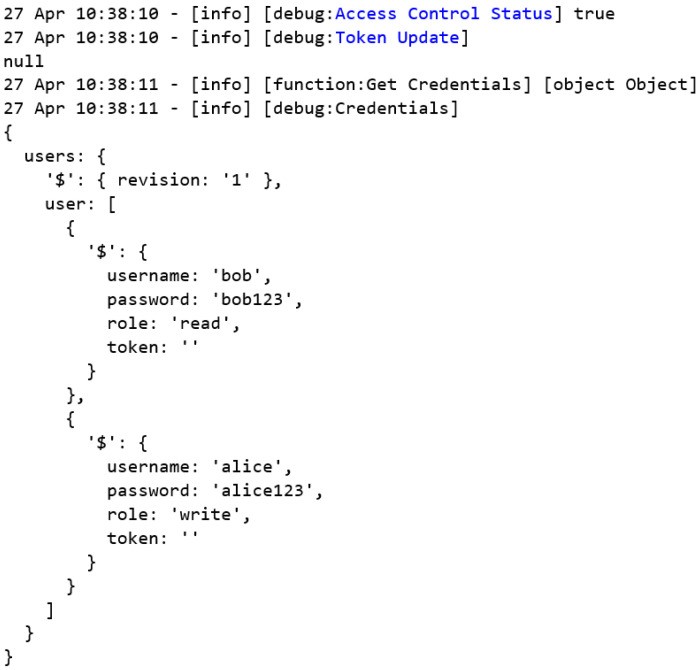
Event log when enabling access control.

When enabling the access control (Figure 16), the global variable token is initially set to null, and the user credentials that were previously stored in the non-volatile memory are loaded into the system.

**Figure 17:**
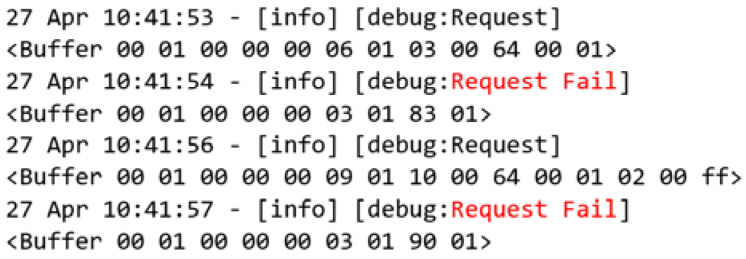
Event log of illegal function to read and write registers.

With the access control enabled, the initial tests of reading and writing in the register 0x64 are repeated (Figure 17) through the read holding register and the write multiple register functions, and it is verified that the Modbus client has its access controlled and denied by the MITM flow. When making requests to the Modbus server register, it receives an illegal function exception for both requests.

**Figure 18:**
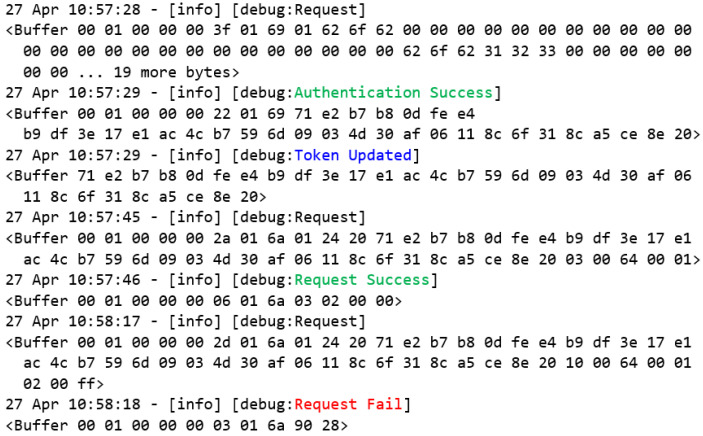
Event log of Bob’s requests.

The following tests will be performed using the new user-defined functions that were developed and proposed in this work. Figure 18 presents the requests that were made by the user that was labeled as Bob. Through the new user-defined function authentication that was proposed by this study (Figure 2a), the MITM flow confirms that Bob is a trusted user and that his credentials are valid before authenticating the user to the system. In this process, a token is generated and registered in the given user’s token key, as presented in Figure 16, and finally returning it in the request-response (Figure 2c).

Once the authentication confirmation has been successfully received, the authentication requests flow configures the global token variable with the token value that is received in the authentication request response, which will be used from now on in the read and write requests that are made through the new user-defined authorization function.

Thus, this performs a new reading request from the Modbus client flow, instead of the standard request through the read holding register function. Once the global token variable value is different from null, the Modbus client flow performs the request using the new proposed function (Figure 3a). This will encapsulate the original read holding register function together with the unique token that identifies the user Bob, allowing the MITM flow to identify the user, originating the given request, and, consequently, allowing access control to the registers and the other resources of the Modbus server by the role of each trusted user in the system.

As shown in Table 1, the user Bob has only the read permission. Therefore, it is evident in this test that it was possible to perform the read operation successfully (Figure 3b). However, when performing the write operation through the encapsulation of the original write multiple register function, the user Bob had his request denied by the MITM flow, returning to the exception according to Figure 3c.

**Figure 19:**
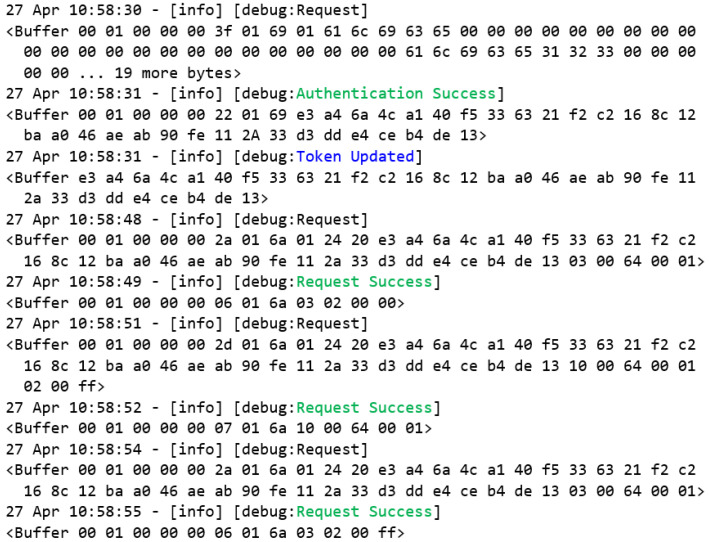
Event log of Alice’s requests.

Figure 19 shows the exact requests that were made by Bob, but now they will be performed by another user, Alice, who, according to Table 1, has both read and write access to the Modbus server registers. First, the user Alice successfully authenticates to the MITM flow, and her respective token is updated to the global token variable. Then, a read request is performed successfully, and a written request is also performed successfully, changing the value of the register to 0xFF. The proof of this change is validated by a second read request that proves that the register 0x64 had its value changed to 0xFF.

**Figure 20:**
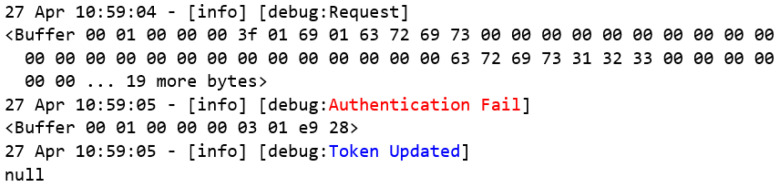
User Cris’s authentication event log.

Finally, Figure 20 presents the logs of a test that was carried out with the untrusted user Cris. According to Table 1, this user is unknown by the system, so when trying to authenticate, the MITM flow returns an exception, and consequently, the global token variable is forced to have a null value.

## 6. Discussion

The IEC 62443-4-2 standard qualifies the security maturity that was reached by a device through its four security levels. Through the implementation and the tests with the developed functions that have been described here, its applicability for constructing the access control system that allows level one of maturity is evidenced. Table 2 presents these items that make up the fundamental requirements for the authentication (FR 1) and the usage control (FR 2). These secure the component against casual attacks by unauthorized users with a generic knowledge of industrial systems, which use simple means, few resources, and low motivation [21].

The Modbus/TCP Security protocol specifies an X.509 certificate attribute in order to define the role of a particular Modbus client device. However, it is the user’s responsibility to build the access control system in order to use such information.

In the implementation that has been presented in this work, the credentials and the access rules are managed natively by the component, the support changes, and new accounts, and enforce the security policies. The new functions that have been developed, in addition to guaranteeing non-repudiation, allow the use of credentials based on a username and a password, which, together with the use of certificates that are specified by the protocol, could create an additional factor of authentication to the system.

Although public key infrastructure certificates are not used during testing, they are essential for the functions to work correctly. Furthermore, they must support revocation or have a limited expiration date in order to ensure encryption security [21].

After identifying and authenticating each user, the usage control through the authorization function protects the component against any unauthorized actions by verifying that the necessary privileges have been granted before allowing a user to perform such actions.

The audit-relevant events with access control timestamps are registered, and their storage would allow future audits to be carried out, which is a capability that was not applied to the tests since, as well as encryption, they were not of paramount importance for validating the developed functions.

Regarding the vulnerabilities of the proposed method, in order to guarantee access to the equipment, it is also necessary to control the other interfaces of access to the device, whether for human users or not, as well as to ensure the quality of the individual secrecy of password-based authenticators, which can be shared between system operators. Therefore, policies must be built in order to limit the authenticate lifetime, forcing operators to use and update them, as well as their responsibility under the credential.

The implemented access control also has vulnerabilities for actors with a lot of resources and knowledge in IACS. In order to mitigate such vulnerabilities, and to guarantee the device integrity through the root of trust and a secure boot process, multifactor authentication capabilities must be employed to all access interfaces. In addition, double approval by supervisors for changing the critical parameters in the system, the non-repudiation for all types of users, the protection of diagnostics interfaces, the automatic notification of integrity violations, the validation of the input data ensuring that they are within a valid range, the deterministic output taking the equipment to a safe state in case of violations, and protections against a denial of service due to a lack of device resources caused by high processing consumption allow control to be applied through the system’s data input and output interfaces, instead of being performed during the establishment of the communication session between devices. This allows the identification and the validation of each human user before providing access to the resources of the device. The protocol improvement approach with authentication and authorization features differs from other published works, as it can be applied directly to the Modbus server, not requiring an additional component. It also makes it possible to extend the access control to the edge of IACS.

Another critical point is that the essential characteristics of the Modbus protocol were not changed. Limiting the original PDU to 216 bytes when encapsulating the new authorization function was necessary. Despite this, as it is a user-defined function, care must be taken to not apply them together with PDUs that extrapolate such an amount of data, which, in the author’s opinion, is feasible since most Modbus public functions do not exceed such a limit.

Finally, the implementation made it possible to validate and exemplify the usability of the functions that have been developed, and the tests demonstrate the segmentation of access by the users existing in the system, ensuring that the users with lower access privileges do not perform the specific operations of the users with higher privileges.

## 7. Conclusions

Industrial networks are no longer physically isolated and are now integrated with corporate intranets and the internet. Due to Industry 4.0 and digital transformation, this shift is taking place abruptly while the Zero Trust concept has replaced traditional IT architectures, such as edge or perimeter control.

The Modbus/TCP Security protocol, which was published by the Modbus organization, establishes the adoption of the TLS protocol in order to increase the cybersecurity maturity of the Modbus/TCP protocol, ensuring communication channel confidentiality and device authentication if the mutual authentication feature is implemented. With the two new user-defined functions that have been proposed in this work, the conclusion is that they allow the enhancement of the protocol, adding authentication and authorization functions that, together with the proposed method, permit the creation of access control for any users (including humans, systems, or devices).

The tests implementing the functions and the access control method through the Node-RED software prove the potential and the applicability of the two new functions. The construction of the access controls, along with the authentication support, ensures the non-repudiation of human users and guarantees security against casual unauthorized access and use attempts of actors with generic knowledge in IACS, using simple means, low level of resources, and low motivation.

This approach allows the protocol to extend the functions that have been developed and the method that has been used to other applications and devices that use client–server industrial communication protocols. This work has presented the construction of access control for a PLC. Nevertheless, an IACS is made up of other equipment, such as power converters. These devices lack safety features and are usually installed in critical infrastructures. Implementing a similar approach in these converters would make it possible to control the access to the users, whether they are humans, devices, or systems, to these devices.

Once cybersecurity in power electronics is one of the authors’ research lines, future research will aim to improve the functions and the methodology that have been proposed in this work in order to implement a robust role-based access control that complies with the second level of IEC 62443-4-2; additionally for those that are embed in a power converter that is applied to electrical drives, which today are increasingly connected and are, consequently, more exposed to cyber-attacks.

## Figures and Tables

**Table 1:** Users’ permissions.

User	Trusted	Write	Read
Alice	Yes	Yes	Yes
Bob	Yes	No	Yes
Cris	No	No	No

**Table 2:** Security level one component requirements (CRs) for FR 1 and FR 2.

Item	Requirement	Description	SL 1
FR 1	CR 1.1	Human user identification and authentication	Yes
FR 1	CR 1.3	Authenticator management	Yes
FR 1	CR 1.4	Identifier management	Yes
FR 1	CR 1.5	Authenticator management	Yes
FR 1	CR 1.7	Strength of password-based authentication	Yes
FR 1	CR 1.10	Authenticator feedback	Yes
FR 1	CR 1.11	Unsuccessful login attempts	Yes
FR 1	CR 1.12	System use notification	NA
FR 2	CR 2.1	Authorization enforcement	Yes
FR 2	CR 2.2	Wireless use control	NA
FR 2	CR 2.5	Session lock	Yes
FR 2	CR 2.8	Auditable events	Yes
FR 2	CR 2.9	Auditable storage capacity	NA
FR 2	CR 2.10	Responses to audit processing failures	NA
FR 2	CR 2.11	Timestamps	Yes
FR 2	CR 2.12	Non-repudiation	Yes
